# Purging frequency and number of purging methods as indicators of severity in bulimia nervosa: Interactive effects with binge eating episodes

**DOI:** 10.1002/erv.3147

**Published:** 2024-10-26

**Authors:** Anna L. Dieffenbacher, Adrian Meule, Ulrich Voderholzer

**Affiliations:** ^1^ Department of Psychiatry and Psychotherapy University Hospital LMU Munich Munich Germany; ^2^ Schoen Clinic Roseneck Prien am Chiemsee Germany; ^3^ Department of Psychology University of Regensburg Regensburg Germany; ^4^ Department of Psychiatry and Psychotherapy University Hospital of Freiburg Freiburg Germany

**Keywords:** binge eating, bulimia nervosa, psychopathology, purging, severity

## Abstract

**Objective:**

In the Diagnostic and Statistical Manual of Mental Disorders‐5, severity of bulimia nervosa (BN) is defined by the frequency of purging behaviour. Previous research suggests the number of purging methods as an alternative rating. The current analysis investigated characteristics (sociodemographic and treatment‐related variables, body mass index [BMI], eating disorder‐specific and general psychopathology) of persons with BN as a function of purging frequency and number of purging methods in order to examine which approach might be better suited for indicating severity of BN.

**Method:**

Two‐hundred and sixty‐one persons (98.5% female; mean age 25.2 years, SD = 9.41; mean BMI 22.0 kg/m^2^, SD = 3.79) with BN completed self‐report questionnaires on eating disorder‐specific and general psychopathology at admission to inpatient (*n* = 214) or daypatient (*n* = 47) treatment.

**Results:**

Higher severity based on either purging frequency or number of purging methods tended to relate to lower BMI and higher eating disorder‐specific and general psychopathology. In addition, binge‐eating frequency differentially related to eating disorder‐specific and general psychopathology as a function of severity groups.

**Conclusions:**

This study partially supports the utility of both purging frequency and the number of purging methods as indicators of severity in persons with BN. However, focussing only on purging behaviours may be short‐sighted as it appears necessary to consider the number of binge‐eating episodes as well when evaluating severity of BN.

## Key points

- The number of purging methods used (instead of the frequency of different purging behaviours combined) has been suggested as an alternative marker of severity of bulimia nervosa.
- This study partially supports the utility of both purging frequency and the number of purging methods as indicators of severity in persons with bulimia nervosa (BN).
- Focussing only on purging behaviours may be short‐sighted as it appears necessary to consider the number of binge‐eating episodes as well when evaluating severity of BN.

## 1 INTRODUCTION

### 1.1 Bulimia nervosa (BN)

BN is an eating disorder characterised by the consumption of large amounts of food in a distinct time period with an associated subjective feeling of loss of control (binging), followed by engagement in compensatory behaviours (e.g., self‐induced vomiting, misuse of laxatives, diuretics or other medication, fasting, excessive exercise) to prevent weight gain or facilitate weight loss. A recent review reported a 3% lifetime prevalence for females and a 1% lifetime prevalence for males (Van Eeden et al., 2021). The most common treatment settings for BN are outpatient, daypatient, and residential/inpatient programs. Each setting has its advantages and may be recommended based on the severity of the condition, patient preferences, and available resources (American Psychiatric Association, 2006).

Medical complications in persons with BN primarily occur due to compensatory behaviours such as vomiting. These can include irritation and inflammation of the oesophagus, gastrointestinal issues such as acid reflux, oesophagitis, and peptic ulcers, electrolyte imbalances, dehydration, dental issues, metabolic changes, musculoskeletal problems, and cardiovascular complications (Gibson et al., 2019; Nitsch et al., 2021; Weigel et al., 2019). As a result, persons with BN report impairment due to stomach pain and nausea, gas, or indigestion as well as more sick leave days (Weigel et al., 2019).

### 1.2 Diagnostic and Statistical Manual of Mental Disorders (DSM‐5) severity indicators

In the DSM‐5 (American Psychiatric Association, 2013), the prior classification between purging and non‐purging subtypes was eliminated and a new severity specifier was added. The severity rating of BN is based on the frequency of purging behaviours: mild (an average of 1–3 purging episodes per week), moderate (an average of 4–7 purging episodes per week), severe (an average of 8–13 purging episodes per week), and extreme (an average of 14 or more purging episodes per week).

A severity specifier should enable clinicians to rate the clinical severity of a certain diagnosis. Specifically, ratings should provide information in terms of intensity, frequency, duration, symptom count, or other disorder‐specific markers of severity (American Psychiatric Association, 2013). Accordingly, severity categories should also reflect meaningful differences in terms of general psychopathology, distress, and prognosis. However, studies investigating the clinical utility of the DSM‐5 severity specifier for BN have been inconsistent (Dang et al., 2022). For example, only small differences in eating disorder pathology and depression between patients with extreme severity and other severity groups have been found (Gianini et al., 2017; Grilo et al., 2015). This finding is in line with other studies (Jenkins et al., 2016; Krug et al., 2021) that question if the severity specifier detects differences on cross‐sectional measures of pathology and its ability to differentiate between the moderate and the severe group.

### 1.3 Number of purging methods as an alternative severity indicator

The number of purging methods used (instead of the frequency of different purging behaviours combined) has been suggested as an alternative marker of severity (Dang et al., 2022). Some studies (Eddy et al., 2009; Edler et al., 2007; Favaro et al., 2005) found that persons who report multiple methods of purging exhibit greater eating disorder pathology, anxiety, self‐injurious behaviour, and suicide attempts compared to persons who report only one purging method. These differences even maintained at 6‐year follow‐up (Ackard et al., 2011) and at 10‐year follow‐up (Haedt et al., 2006). Furthermore, in a sample of 93 patients with BN, patients with multiple purging methods had a significantly higher level of eating and weight concerns than those in the single purging method group (Gianini et al., 2017). From the medical side, it has also been noted that electrolyte disturbance might be more severe because of the use of multiple purging methods (Nitsch et al., 2021). Thus, there is initial support for the number of purging methods as an alternative to purging frequency as a severity indicator in persons with BN.

### 1.4 The role of binge‐eating frequency in BN severity

Both approaches outlined above (purging frequency and number of purging methods) only capture one facet of BN (purging) but do not consider other core features such as drive for thinness (Krug et al., 2021). In addition, while inappropriate compensatory behaviour is the core feature of BN, it is typically preceded by binge‐eating episodes (American Psychiatric Association, 2013). However, the role of binge‐eating frequency has rarely been considered in the discussion about severity indicators of BN. This comes as a surprize as higher binge‐eating frequency relates to higher eating disorder‐specific and general psychopathology as well as to lower life satisfaction (Guerdjikova et al., 2019; Reichborn‐Kjennerud et al., 2004).

In persons with BN, it might be that correlates of binge‐eating frequency differ as a function of purging frequency or the number of purging methods used. For example, it appears that negative affect is increased after a binge‐eating episode but subsequent purging decreases this negative affect (Haedt‐Matt & Keel, 2011). Thus, it might be speculated that when binge eating and purging diverges (e.g., when a person binges without subsequent purging or purges without having previously binged), this may also affect psychopathological correlates (e.g., lower life satisfaction due to weight gain when binges are not compensated by purging).

### 1.5 The current study

The aims of the current study were threefold. First, we examined whether higher BN severity based on purging frequency or based on the number of purging methods used would be associated with higher eating disorder‐specific and general psychopathology. Second, we examined whether binge‐eating frequency would be associated with higher eating‐disorder specific and general psychopathology. Third, we examined interactive effects between BN severity (based on either purging frequency or on the number of purging methods) and binge‐eating episodes on eating disorder‐specific and general psychopathology.

## 2 METHODS

### 2.1 Sample description

**FIGURE 1:**
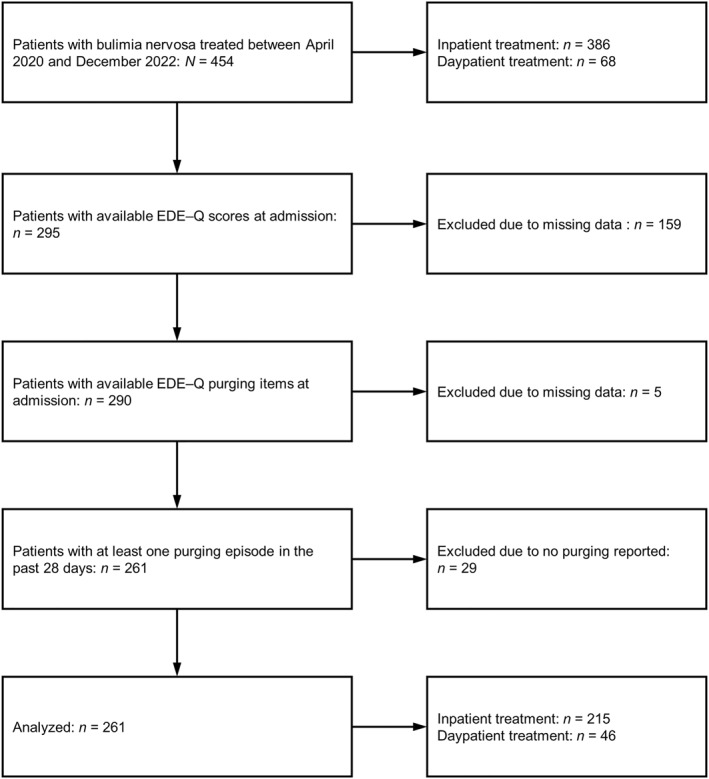
Flowchart of patients included in the analyses. EDE‐Q, Eating Disorder Examination‐Questionnaire.

Clinical records of patients with BN (*N* = 261) on admission to inpatient (*n* = 214) or daypatient (*n* = 47) treatment at the Schoen Clinic Roseneck (Prien am Chiemsee) between 2020 and 2022 were analysed (Figure 1). The treatment at the hospital adheres to the German S3‐guidelines for the treatment of BN in terms of admission criteria, treatment elements, and therapy goals (Herpertz et al., 2019). While the majority of persons with BN can be treated in an outpatient setting, inpatient or daypatient treatment is indicated when previous outpatient treatment has been unsuccessful or when other circumstances require a more intensive treatment setting (e.g., substantial mental and physical comorbidity, high illness severity). At the hospital, the day‐ and inpatient treatment is quite similar except that patients in the daypatient programme do not eat dinner at the hospital and do not stay overnight and over the weekend. In both programs, patients received a cognitive behaviour therapy‐oriented, multimodal BN treatment that included several treatment elements such as individual psychotherapy sessions, group therapy sessions, supervised meals, exercise therapy, meal preparation classes, body image exposure, nutrition counselling, food intake protocols, and clinical management of medical complications.

At the hospital, data from the diagnostic assessments (e.g., age, sex, height, weight, ICD‐10‐based diagnoses, questionnaire scores) are automatically transferred to a database from which they can be exported without any identifying information (e.g., name, date of birth, place of residence) by authorised employees. Thus, accessing individual patient charts is not necessary. According to the guidelines by the ethics committee of the LMU Munich, retrospective studies conducted on already available, anonymised data are exempt from requiring ethics approval. Inclusion criteria were (1) a diagnosis of either full syndrome or atypical BN according to the 10th version of International Classification of Diseases (ICD‐10 codes F50.2 and F50.3), (2) available Eating Disorder Examination‐Questionnaire (EDE‐Q) scores at admission, and (3) at least one self‐reported purging episode in the past 28 days. The majority of patients were diagnosed with full syndrome BN (85.1%, *n* = 222; atypical BN: 14.9%, *n* = 39) and were female (98.5%, *n* = 257; male: 1.5%, *n* = 2). One‐hundred and thirteen patients (43.4%) had at least one comorbid mental disorder. The most common comorbid mental disorders were affective disorders (ICD‐10 code: F3; 36.4%; *n* = 95). Mean age was 25.2 years (SD = 9.41; adolescents [<18 years]: 21.5%, *n* = 56) and mean body mass index (BMI) at admission was 22 kg/m^2^ (SD = 3.79).

At admission, patients reported self‐induced vomiting *M* = 19.8 times (SD = 22.12), use of laxatives *M* = 1.0 times (SD = 3.93), and excessive exercise *M* = 6.6 times (SD = 9.14) in the past 28 days as assessed with the EDE‐Q. When computing the average frequency of purging behaviours used per week (by summing up all numbers of times of all three behaviours and dividing them by four), 82 patients (31.4%) would be categorised as mild, 99 patients (37.9%) as moderate, 48 patients (18.4%) as severe, and 32 patients (12.3%) as extreme, according to the DSM‐5 severity specifier. When grouping the sample according to the number of purging methods, 124 patients (47.5%) reported using one purging method (i.e., at least one purging episode for either vomiting, using laxatives, or excessive exercising), 115 patients (44.1%) reported using two purging methods (i.e., at least one purging episode for at least two purging behaviours), and 22 patients (8.4%) reported using three purging methods (i.e., at least one purging episode for all three purging behaviours) in the past 28 days as assessed with the EDE‐Q.

### 2.2 Measures

#### 2.2.1 EDE‐Q

Eating disorder symptoms were measured with the German version (Hilbert et al., 2007) of the EDE‐Q (Fairburn & Beglin, 1994). The EDE‐Q consists of 28 items, six of which assess the frequency of purge and binge behaviours within the last 28 days and are not included in the total score. Specifically, items 13 and 14 ask about the number of times consuming large amounts of food and experiencing a feeling of loss of control, priming item 15, which assesses the number of binge days (‘Over the past 28 days, on how many DAYS have such episodes of overeating occurred?’), item 16 assesses the number of times of self‐induced vomiting (‘Over the past 28 days, how many times have you made yourself sick [vomit] as a means of controlling your shape or weight?’), item 17 assesses the number of times using laxatives (‘Over the past 28 days, how many times have you taken laxatives as a means of controlling your shape or weight?’), and item 18 assesses the number of times exercising excessively (‘Over the past 28 days, how many times have you exercised in a “driven” or “compulsive” way as a means of controlling your weight, shape or amount of fat, or to burn off calories?’). The remaining 22 items measure eating restraint, eating concern, weight concern, and shape concern and responses are recorded on a seven‐point scale with different response labels (coded with 0–6). Higher mean scores indicate higher eating disorder symptomatology. As the four‐factor structure of the EDE‐Q could rarely be replicated (Rand‐Giovannetti et al., 2020), we only analysed the total score in the current study. Internal reliability was *ω* = 0.916 in the current study.

#### 2.2.2 Compulsive Exercise Test (CET)

Compulsive exercise was measured with the German version (Schlegl et al., 2022) of the CET (Taranis et al., 2011). The CET consists of 24 items and assesses avoidance and rule‐driven behaviour, weight control exercise, mood improvement, lack of exercise enjoyment, and exercise rigidity. Responses are recorded on a six‐point scale from 0 (never true) to 5 (always true). Higher scores indicate a greater degree of compulsive exercise (Schlegl et al., 2022). Internal reliability was *ω* = 0.941 in the current study.

#### 2.2.3 Patient Health Questionnaire–Depressive Symptom Severity Scale (PHQ‐9)

Depressive symptoms were measured with the German version (Löwe et al., 2002) of the PHQ‐9 (Kroenke et al., 2001; Kroenke & Spitzer, 2002). The PHQ‐9 consists of nine items and assesses nine core aspects of depression including affective, cognitive, and somatic symptoms. Items responses are recorded on a four‐point scale ranging from 0 (not at all) to 3 (nearly every day). Higher scores indicate higher depressive symptom severity. Internal reliability was *ω* = 0.843 in the current study.

#### 2.2.4 Patient Health Questionnaire–Somatic Symptom Severity Scale (PHQ‐15)

Somatic symptoms were measured with the German version (Löwe et al., 2002) of the PHQ‐15 (Kroenke et al., 2002). The PHQ‐15 consists of 15 items and assesses the burden of common somatic symptoms. Responses are recorded on a three‐point scale that ranges from 0 (not bothered at all) to 2 (bothered a lot). Higher scores indicate higher somatic symptom severity. Internal reliability was *ω* = 0.750 in the current study.

#### 2.2.5 Generalised Anxiety Disorder Scale (GAD‐7)

Anxiety symptoms were measured with the German version (Löwe et al., 2002) of the GAD‐7 (Spitzer et al., 2006). The GAD‐7 consists of seven items and assesses the core symptoms of generalised anxiety disorder. Responses are recorded on a four‐point scale that ranges from 0 (never) to 3 (almost every day). Higher scores indicate higher anxiety symptom severity. Internal reliability was *ω* = 0.819 in the current study.

#### 2.2.6 Satisfaction With Life Scale (SWLS)

Life satisfaction was measured with the German version (Glaesmer et al., 2011) of the SWLS (Diener et al., 1985). The SWLS consists of five items and assesses overall satisfaction with life as a whole. Responses are recorded on a seven‐point scale from 1 (strongly disagree) to 7 (strongly agree). Higher scores indicate higher life satisfaction. Internal reliability was *ω* = 0.843 in the current study.

### 2.3 Data analyses

Data were analysed with JASP version 0.16.4.0 (JASP Team, 2023) and R 4.2.1 (RCore Team, 2022) in RStudio 2022.07.1 (RStudio Team, 2022). As only 32 patients were classified as having extreme severity according to purging frequency, we combined the severe and extreme group, thus comparing three groups (mild vs. moderate vs. severe/extreme) based on purging frequency. Similarly, as only 22 patients reported using three purging methods, we combined patients reporting two and three purging methods, thus comparing two groups (one vs. multiple purging methods) based on the number of purging methods. As assumptions of the general linear model are often violated when analysing psychological data in general and clinical psychology data in particular, it has been suggested to prefer non‐parametric and robust analysis techniques (Field & Wilcox, 2017). Therefore, groups were compared regarding continuous variables (i.e., age, BMI, EDE‐Q scores, CET scores, PHQ‐9 scores, PHQ‐15 scores, GAD‐7 scores, SWLS scores) with Kruskal–Wallis tests and Dunn's tests for post‐hoc comparisons (purging frequency groups) and with Mann–Whitney–U tests (number of purging methods groups). Groups were compared regarding categorical variables (treatment type, BN subtype, any comorbidity, sex) with Fisher's exact tests.

Based on the recommendation of one of the reviewers, we further examined differences in eating disorder‐specific and general psychopathology between groups when controlling for sociodemographic and treatment‐related variables. For this, we used robust regressions (Koller & Stahel, 2011) with the R package *robustbase* (Maechler et al., 2023). Specifically, severity groups along with age, BMI, treatment type, BN subtype, any comorbidity, and sex were used as independent variables and EDE‐Q scores, CET scores, PHQ‐9 scores, PHQ‐15 scores, GAD‐7 scores, and SWLS scores as dependent variables. As there were three purging frequency groups, we used indicator coding to create two dummy‐coded variables (Hayes & Montoya, 2017) using the moderate severity group as reference. That is, the two dummy‐coded variables thus represent comparisons of the mild versus moderate severity group and the moderate versus severe/extreme severity group.

Relationships of the number of binge days with eating disorder psychopathology, compulsive exercise, depressive symptoms, somatic symptoms, anxiety symptoms, and life satisfaction were tested with robust percentage bend correlation coefficients (Wilcox, 1994) with the R package *WRS2* (Mair & Wilcox, 2020). Interaction effects between the number of binge days and severity groups on eating disorder‐specific and general psychopathology were tested with robust regressions (Koller & Stahel, 2011) with the R package *robustbase* (Maechler et al., 2023). Specifically, separate models were calculated with the independent variables groups, binge days, and groups × binge days and either the dependent variables eating disorder psychopathology, compulsive exercise, depressive symptoms, somatic symptoms, anxiety symptoms, or life satisfaction. Note that the purging frequency groups are represented by two dummy‐coded variables (thus producing two interaction terms; Hayes & Montoya, 2017), as outlined above. Significant interaction effects were probed by examining percentage bend correlation coefficients between binge days and the given dependent variable within each group.

For interpretation of all inferential tests, we used an alpha level of 0.050, that is, we did not adjust for multiple testing. The reason for this is that it is unclear which alternative value should be chosen (e.g., by which number of tests the alpha value should be divided and if this procedure should be used in the first place) and that lowering the alpha level substantially increases the risk for Type II error (Perneger, 1998). Under certain circumstances, choosing a smaller alpha level even leads to a much higher error rate when considering both Type I and Type II error combined than when using an alpha level of 0.050 (Witt, 2019). The data and R code for the correlation and regression analyses can be accessed at https://osf.io/khb5r.

## 3 RESULTS

### 3.1 Differences between severity groups

Purging frequency groups differed in BMI, eating disorder psychopathology, binge days, vomiting frequency, use of laxatives, frequency of excessive exercise, compulsive exercise, depressive symptoms, somatic symptoms, and anxiety symptoms (Table 1). Post‐hoc tests revealed that the mild severity group had a higher BMI, lower eating disorder psychopathology, fewer binge days, lower vomiting frequency, lower use of laxatives, lower frequency of excessive exercise, lower compulsive exercise scores, less depressive symptoms, less somatic symptoms, and less anxiety symptoms than the moderate and the severe/extreme groups (all *p*s < 0.05). Patients in the moderate severity group had lower eating disorder psychopathology, fewer binge days, lower vomiting frequency, lower frequency of excessive exercise, lower compulsive exercise scores, and less depressive symptoms than patients in the severe/extreme severity group (all *p*s < 0.05). The differences in eating disorder‐specific and general psychopathology partially remained significant when controlling for sociodemographic and treatment‐related variables (Table 2).

**TABLE 1:** Descriptive and test statistics of study variables as a function of purging frequency groups.

	Mild severity	Moderate severity	Severe/extreme severity	Test statistics
Sex (female)	*n* = 82 (100%)	*n* = 96 (97%)	*n* = 79 (98.8%)	*χ* ^2^ = 2.79, *p* = 0.248, *V* = 0.10
Bulimia nervosa subtype (full syndrome, F50.2)	*n* = 64 (78%)	*n* = 87 (87.9%)	*n* = 71 (88.8%)	*χ* ^2^ = 4.65, *p* = 0.098, *V* = 0.13
Any comorbidity (yes)	*n* = 50 (61%)	*n* = 96 (97%)	*n* = 51 (63.8%)	*χ* ^2^ = 1.60, *p* = 0.499, *V* = 0.08
Treatment type (inpatient)	*n* = 61 (74.4%)	*n* = 83 (83.8%)	*n* = 70 (87.5%)	*χ* ^2^ = 5.08, *p* = 0.079, *V* = 0.14
Age (years)	*n* = 82, *M* = 26.24, SD = 11.22	*n* = 99, *M* = 24.79, SD = 8.56	*n* = 80, *M* = 24.53, SD = 8.33	*H*_(2)_ = 0.14, *p* = 0.935, *η* ^2^ = 0.01
Body mass index (kg/m^2^)	*n* = 82, *M* = 22.91, SD = 4.50	*n* = 99, *M* = 21.56, SD = 3.18	*n* = 80, *M* = 21.77, SD = 3.57	*H*_(2)_ = 8.15, *p* = 0.017, *η* ^2^ = 0.02
Eating Disorder Examination‐Questionnaire				
Global score	*n* = 82, *M* = 3.76, SD = 1.23	*n* = 99, *M* = 4.17, SD = 1.19	*n* = 80, *M* = 4.54, SD = 0.98	*H*_(2)_ = 19.05, *p* < 0.001, *η* ^2^ = 0.07
Binge days	*n* = 81, *M* = 8.99, SD = 7.75	*n* = 99, *M* = 16.14, SD = 9.35	*n* = 80, *M* = 19.41, SD = 9.41	*H*_(2)_ = 46.26, *p* < 0.001, *η* ^2^ = 0.19
Vomiting	*n* = 82, *M* = 3.73, SD = 3.67	*n* = 99, *M* = 16.60, SD = 9.49	*n* = 80, *M* = 40.36, SD = 28.06	*H*_(2)_ = 139.00, *p* < 0.001, *η* ^2^ = 0.44
Laxatives	*n* = 82, *M* = 0.22, SD = 0.86	*n* = 99, *M* = 0.34, SD = 0.98	*n* = 80, *M* = 2.48, SD = 6.74	*H*_(2)_ = 7.27, *p* = 0.026, *η* ^2^ = 0.07
Compulsive exercise	*n* = 82, *M* = 2.56, SD = 3.25	*n* = 99, *M* = 6.11, SD = 8.76	*n* = 80, *M* = 11.39, SD = 11.33	*H*_(2)_ = 31.46, *p* < 0.001, *η* ^2^ = 0.15
Compulsive Exercise Test	*n* = 81, *M* = 2.27, SD = 0.72	*n* = 93, *M* = 2.44, SD = 1.01	*n* = 79, *M* = 2.87, SD = 0.91	*H*_(2)_ = 19.27, *p* < 0.001, *η* ^2^ = 0.07
Patient Health Questionnaire‐9	*n* = 76, *M* = 13.61, SD = 5.59	*n* = 89, *M* = 15.99, SD = 5.67	*n* = 75, *M* = 17.49, SD = 5.40	*H*_(2)_ = 8.15, *p* < 0.001, *η* ^2^ = 0.07
Patient Health Questionnaire‐15	*n* = 76, *M* = 10.84, SD = 5.35	*n* = 89, *M* = 12.67, SD = 4.72	*n* = 75, *M* = 12.95, SD = 4.94	*H*_(2)_ = 18.21, *p* = 0.025, *η* ^2^ = 0.03
Generalised Anxiety Disorder‐7	*n* = 76, *M* = 9.37, SD = 4.69	*n* = 89, *M* = 11.46, SD = 5.24	*n* = 75, *M* = 12.10, SD = 4.03	*H*_(2)_ = 7.39, *p* = 0.001, *η* ^2^ = 0.06
Satisfaction With Life Scale	*n* = 75, *M* = 16.59, SD = 6.46	*n* = 91, *M* = 16.41, SD = 6.97	*n* = 75, *M* = 15.19, SD = 6.44	*H*_(2)_ = 2.47, *p* = 0.291, *η* ^2^ = 0.01

**TABLE 2:** Unstandardised coefficients of robust regression models for the effect of purging frequency groups on eating disorder‐specific and general psychopathology with covariates.

Independent variables	Dependent variables																	
	Eating Disorder Examination‐Questionnaire			Compulsive Exercise Test			Patient Health Questionnaire‐9			Patient Health Questionnaire‐15			Generalised Anxiety Disorder‐7			Satisfaction With Life Scale		
	*b*	SE	*p*	*b*	SE	*p*	*b*	SE	*p*	*b*	SE	*p*	*b*	SE	*p*	*b*	SE	*p*
Intercept	3.58	0.40	<0.001	2.40	0.41	<0.001	16.81	2.31	<0.001	10.50	2.62	<0.001	12.99	2.00	<0.001	14.43	2.95	<0.001
D1	−0.37	0.19	0.048	−0.20	0.15	0.176	−1.70	0.90	0.061	−1.73	0.89	0.052	−1.53	0.82	0.061	−0.52	1.07	0.629
D2	0.32	0.16	0.053	0.48	0.16	0.004	2.04	0.84	0.016	0.38	0.81	0.635	1.03	0.73	0.161	−1.79	1.02	0.080
Age (years)	−0.02	0.01	0.007	−0.01	0.01	0.093	−0.12	0.04	0.005	0.03	0.04	0.502	−0.07	0.03	0.007	0.14	0.04	<0.001
Sex	0.39	1.54	0.801	−0.83	0.77	0.285	1.59	4.86	0.744	−2.76	2.34	0.240	1.67	0.33	0.780	−3.22	6.40	0.615
Treatment type	−0.42	0.23	0.072	0.20	0.14	0.161	−1.99	0.97	0.042	−0.73	0.95	0.444	−0.90	0.84	0.280	2.15	0.99	0.031
Any comorbidity	0.38	0.16	0.015	0.28	0.12	0.021	3.83	0.86	<0.001	1.46	0.78	0.061	3.15	0.65	<0.001	−5.16	0.95	<0.001
Bulimia nervosa subtype	0.00	0.20	0.992	0.27	0.15	0.079	−0.20	1.01	0.841	−0.33	0.87	0.705	0.64	0.84	0.447	0.50	1.24	0.688
Body mass index (kg/m^2^)	0.05	0.02	0.006	0.01	0.02	0.782	−0.02	0.08	0.818	0.03	0.11	0.807	−0.09	0.08	0.251	0.07	0.14	0.602

*Note*: Categorical variables were coded as follows: groups: D1 = Dummy‐coded variable for moderate versus mild severity; D2 = Dummy‐coded variable for moderate versus severe/extreme severity; sex: 0 = female, 1 = male; treatment type: 0 = inpatient, 1 = daypatient; comorbidity: 0 = no, 1 = yes; bulimia nervosa subtype: 0 = typical, 1 = atypical.

Number of purging methods groups differed in age, BMI, eating disorder psychopathology, purging, and anxiety symptoms such that patients in the multiple purging methods group were younger, had a lower BMI, had higher eating disorder psychopathology, higher vomiting frequency, higher use of laxatives, higher frequency of excessive exercise, higher compulsive exercise scores, and more anxiety symptoms than patients in the one purging method group (Table 3). The differences in eating disorder‐specific and general psychopathology remained significant when controlling for sociodemographic and treatment‐related variables (Table 4).

**TABLE 3:** Descriptive and test statistics of study variables as a function of number of purging methods groups.

	One purging method	Multiple purging methods	Test statistics
Sex (female)	*n* = 121 (97.6%)	*n* = 136 (99.3%)	*χ* ^2^ = 1.23, *p* = 0.27, *V* = 0.07
Bulimia nervosa subtype (full syndrome, F50.2)	*n* = 101 (81.5%)	*n* = 121 (88.3%)	*χ* ^2^ = 2.42, *p* = 0.12, *V* = 0.10
Any comorbidity (yes)	*n* = 81 (47.6%)	*n* = 89 (65%)	*χ* ^2^ < 0.01, *p* = 0.95, *V* < 0.01
Treatment type (inpatient)	*n* = 101 (65.3%)	*n* = 113 (82.5%)	*χ* ^2^ = 0.05, *p* = 0.83, *V* = 0.01
Age (years)	*n* = 124, *M* = 27.37, SD = 10.5	*n* = 137, *M* = 23.17, SD = 7.78	*U* = 10,621, *p* < 0.001, *r*_rb_ = 0.25 [0.12; 0.38]
Body mass index (kg/m^2^)	*n* = 124, *M* = 22.62, SD = 4.34	*n* = 137, *M* = 21.53, SD = 3.13	*U* = 10,025, *p* = 0.012, *r*_rb_ = 0.18 [0.04; 0.31]
Eating Disorder Examination‐Questionnaire			
Global score	*n* = 124, *M* = 3.90, SD = 1.22	*n* = 137, *M* = 4.38, SD = 1.10	*U* = 6486, *p* < 0.001, *r*_rb_ = −0.24 [−0.36; −0.10]
Binge days	*n* = 124, *M* = 14.90, SD = 9.80	*n* = 136, *M* = 14.94, SD = 9.88	*U* = 8515, *p* = 0.891, *r*_rb_ = 0.01 [−0.13; 0.15]
Vomiting	*n* = 124, *M* = 17.54, SD = 21.16	*n* = 137, *M* = 21.92, SD = 22.98	*U* = 7206, *p* = 0.034, *r*_rb_ = −0.15 [−0.29; −0.01]
Laxatives	*n* = 124, *M* = 0.04, SD = 0.32	*n* = 137, *M* = 1.80, SD = 5.28	*U* = 6272, *p* < 0.001, *r*_rb_ = −0.26 [−0.39; −0.13]
Compulsive exercise	*n* = 124, *M* = 2.50, SD = 6.42	*n* = 137, *M* = 10.34, SD = 9.64	*U* = 2518, *p* < 0.001, *r*_rb_ = −0.70 [−0.77; −0.63]
Compulsive Exercise Test	*n* = 120, *M* = 2.14, SD = 0.87	*n* = 133, *M* = 2.86, SD = 0.85	*U* = 4346, *p* < 0.001, *r*_rb_ = −0.46 [−0.56; −0.34]
Patient Health Questionnaire‐9	*n* = 115, *M* = 14.99, SD = 6.00	*n* = 125, *M* = 16.36, SD = 5.48	*U* = 6222, *p* = 0.072, *r*_rb_ = −0.13 [−0.28; 0.01]
Patient Health Questionnaire‐15	*n* = 115, *M* = 11.70, SD = 5.24	*n* = 125, *M* = 12.62, SD = 4.86	*U* = 6418, *p* = 0.152, *r*_rb_ = −0.11 [−0.25; −0.04]
Generalised Anxiety Disorder‐7	*n* = 115, *M* = 10.14, SD = 5.19	*n* = 125, *M* = 11.79, SD = 4.35	*U* = 5786, *p* = 0.009, *r*_rb_ = −0.20 [−0.33; −0.05]
Satisfaction With Life Scale	*n* = 115, *M* = 16.05, SD = 7.22	*n* = 126, *M* = 16.11, SD = 6.11	*U* = 7088, *p* = 0.772, *r*_rb_ = −0.02 [−0.16; 0.12]

**TABLE 4:** Unstandardised coefficients of robust regression models for the effect of number of purging methods groups on eating disorder‐specific and general psychopathology with covariates.

Independent variables	Dependent variables																	
	Eating Disorder Examination‐Questionnaire			Compulsive Exercise Test			Patient Health Questionnaire‐9			Patient Health Questionnaire‐15			Generalised Anxiety Disorder‐7			Satisfaction With Life Scale		
	*b*	SE	*p*	*b*	SE	*p*	*b*	SE	*p*	*b*	SE	*p*	*b*	SE	*p*	*b*	SE	*p*
Intercept	3.23	0.43	<0.001	1.88	0.41	<0.001	17.04	2.39	<0.001	9.62	2.77	<0.001	12.22	2.11	<0.001	12.80	3.01	<0.001
Groups	0.46	0.15	0.002	0.74	0.11	<0.001	0.94	0.72	0.192	1.07	0.71	0.133	1.45	0.63	0.021	0.79	0.85	0.353
Age (years)	−0.02	0.01	0.016	−0.01	0.01	0.451	−0.11	0.04	0.007	0.04	0.04	0.421	−0.06	0.03	0.030	0.15	0.04	<0.001
Sex	0.45	1.56	<0.001	−0.59	0.67	0.384	1.96	4.64	0.674	−2.17	2.46	0.379	1.70	3.42	0.620	−3.12	6.67	0.640
Treatment type	−0.54	0.23	0.020	0.10	0.14	0.468	−2.57	0.99	0.010	−1.18	0.96	0.217	−1.35	0.88	0.125	2.27	0.97	0.020
Any comorbidity	0.43	0.16	0.007	0.25	0.12	0.031	3.84	0.84	<0.001	1.67	0.77	0.032	3.16	0.64	<0.001	−5.00	0.94	<0.001
Bulimia nervosa subtype	−0.03	0.20	0.862	0.24	0.15	0.119	−0.60	0.99	0.544	0.87	0.87	0.538	0.42	0.82	0.606	0.59	1.26	0.638
Body mass index (kg/m^2^)	0.05	0.02	0.006	0.01	0.02	0.655	−0.05	0.09	0.593	0.01	0.11	0.892	−0.11	0.09	0.219	0.08	0.13	0.540

*Note*: Categorical variables were coded as follows: groups: 0 = one purging method, 1 = two or three purging methods; sex: 0 = female, 1 = male; treatment type: 0 = inpatient, 1 = daypatient; comorbidity: 0 = no, 1 = yes; bulimia nervosa subtype: 0 = typical, 1 = atypical.

### 3.2 Correlates of binge‐eating frequency

More binge days related to higher eating disorder psychopathology (pbcor = 0.148, 95% CI [0.020; 0.270], *p* = 0.017), less compulsive exercise (pbcor = −0.139, 95% CI [−0.266; −0.007], *p* = 0.028), more depressive symptoms (pbcor = 0.189, 95% CI [0.059; 0.311], *p* = 0.003), and lower life satisfaction (pbcor = −0.141, 95% CI [−0.260; −0.018], *p* = 0.029). The number of binge days did not relate to somatic symptoms (pbcor = 0.060, 95% CI [−0.071; 0.185], *p* = 0.354) and anxiety symptoms (pbcor = 0.089, 95% CI [−0.034; 0.222], *p* = 0.172).

### 3.3 Interactive effects between severity groups and binge‐eating frequency

**FIGURE 2:**
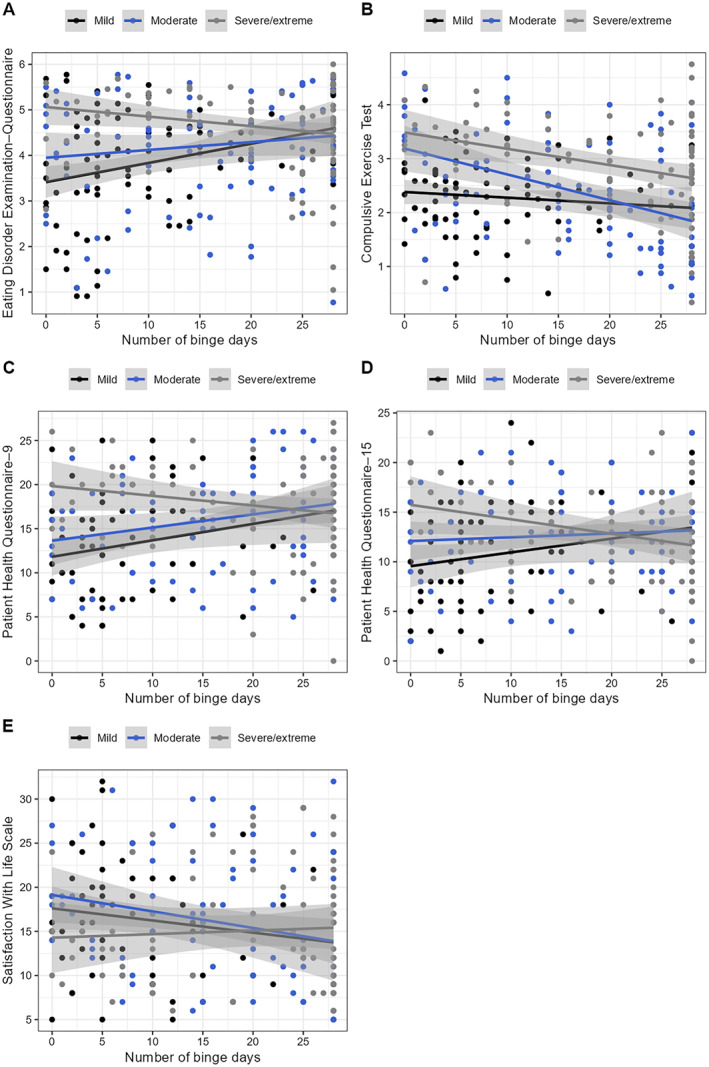
Relationship of binge days with eating disorder psychopathology (a), compulsive exercise (b), depressive symptoms (c), somatic symptoms (d), and life satisfaction (e) as a function of purging frequency groups. The grey‐shaded areas indicate 95% confidence intervals of the robust, linear trend lines.

There were interactive effects between purging frequency groups × binge days when predicting eating disorder psychopathology, compulsive exercise, depressive symptoms, somatic symptoms, and life satisfaction (Table 5). Probing the nature of these interaction effects revealed that more binge days related to higher eating disorder psychopathology in the mild severity group (pbcor = 0.257, 95% CI [0.035; 0.470], *p* = 0.021), did not relate to eating disorder psychopathology in the moderate severity group (pbcor = 0.132, 95% CI [−0.069; 0.331], *p* = 0.194), and to lower eating disorder psychopathology in the severe/extreme severity group (pbcor = −261, 95% CI [−0.468; −0.039], *p* = 0.017; Figure 2a). More binge days related to lower compulsive exercise in the moderate (pbcor = −0.364, 95% CI [−0.555; −0.164], *p* < 0.001) and in the severe/extreme severity group (pbcor = −313, 95% CI [−0.510; −0.079], *p* = 0.005) but were unrelated to compulsive exercise in the mild severity group (pbcor = −0.044, 95% CI [−0.277; 0.159], *p* = 0.695; Figure 2b). More binge days related to more depressive symptoms in the mild (pbcor = 0.268, 95% CI [0.051; 0.460], *p* = 0.020) and in the moderate severity group (pbcor = 0.231, 95% CI [0.016; 0.423], *p* = 0.030) but were unrelated to depressive symptoms in the severe/extreme severity group (pbcor = −0.201, 95% CI [−0.407; 0.016], *p* = 0.084; Figure 2c). More binge days related to fewer somatic symptoms in the severe/extreme severity group (pbcor = −0.249, 95% CI [−0.468; −0.025], *p* = 0.032) but were unrelated to somatic symptoms in the mild (pbcor = 0.202, 95% CI [−0.021; 0.426], *p* = 0.082) and moderate (pbcor = −0.048, 95% CI [−0.163; 0.267], *p* = 0.657; Figure 2d) severity groups. More binge days related to lower life satisfaction in the moderate severity group (pbcor = −0.249, 95% CI [−0.434; −0.046], *p* = 0.018) but were unrelated to life satisfaction in the mild (pbcor = −0.190, 95% CI [−0.406; −0.044], *p* = 0.105) and in the severe/extreme severity group (pbcor = 0.058, 95% CI [−0.169; 0.274], *p* = 0.620; Figure 2e).

**TABLE 5:** Unstandardised coefficients of robust regression models for the interactive effect between purging frequency groups and binge days on eating disorder‐specific and general psychopathology.

Independent variables	Dependent variables																	
	Eating Disorder Examination‐Questionnaire			Compulsive Exercise Test			Patient Health Questionnaire‐9			Patient Health Questionnaire‐15			Generalised Anxiety Disorder‐7			Satisfaction With Life Scale		
	*b*	SE	*p*	*b*	SE	*p*	*b*	SE	*p*	*b*	SE	*p*	*b*	SE	*p*	*b*	SE	*p*
Intercept	4.02	0.30	<0.001	3.22	0.22	<0.001	13.73	1.18	<0.001	12.00	0.98	<0.001	11.04	1.41	<0.001	19.09	1.31	<0.001
D1	−0.61	0.40	0.131	−0.85	0.25	<0.001	−1.83	1.55	0.240	−2.44	1.49	0.453	−2.10	1.66	0.206	−1.43	1.79	0.423
D2	1.02	0.36	0.005	0.29	0.28	0.317	6.11	1.59	0.240	3.78	1.52	0.103	2.03	1.60	0.208	−5.14	1.96	0.009
Binge days	0.02	0.01	0.241	−0.05	0.01	<0.001	0.15	0.07	0.032	0.04	0.06	0.014	0.02	0.07	0.766	−0.19	0.07	0.009
D1 × binge days	0.03	0.02	0.241	0.04	0.02	0.016	0.04	0.10	0.726	0.10	0.16	0.353	0.02	0.10	0.821	0.05	0.13	0.699
D2 × binge days	−0.04	0.02	0.037	0.02	0.02	0.218	−0.26	0.09	0.004	−0.19	0.08	0.017	−0.07	0.09	0.392	0.24	0.10	0.019

*Note*: Categorical variables were coded as follows: D1 = Dummy‐coded variable for mild versus moderate severity; D2 = Dummy‐coded variable for moderate versus severe/extreme severity.

**FIGURE 3:**
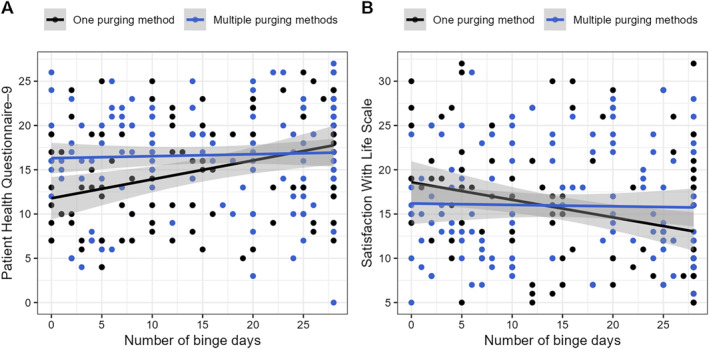
Relationship of binge days with depressive symptoms (a) and life satisfaction (b) as a function of number of purging methods groups. The grey‐shaded areas indicate 95% confidence intervals of the robust, linear trend lines.

There were interactive effects between number of purging methods groups × binge days when predicting depressive symptoms and life satisfaction (Table 6). Probing the nature of these interaction effects revealed that more binge days related to higher depressive symptoms (pbcor = 0.337, 95% CI [0.162; 0.499], *p* < 0.001) and lower life satisfaction (pbcor = −0.251, 95% CI [−0.416; −0.072], *p* = 0.007) in the group with one purging method but not in the group with multiple purging methods (pbcor = 0.041, 95% CI [−0.136; 0.212], *p* = 0.653; pbcor = −0.038, 95% CI [−0.207; 0.131], *p* = 0.678; Figure 3).

**TABLE 6:** Unstandardised coefficients of robust regression models for the interactive effect between number of purging methods groups and binge days on eating disorder‐specific and general psychopathology.

Independent variables	Dependent variables																	
	Eating Disorder Examination‐Questionnaire			Compulsive Exercise Test			Patient Health Questionnaire‐9			Patient Health Questionnaire‐15			Generalised Anxiety Disorder‐7			Satisfaction With Life Scale		
	*b*	SE	*p*	*b*	SE	*p*	*b*	SE	*p*	*b*	SE	*p*	*b*	SE	*p*	*b*	SE	*p*
Intercept	3.60	0.23	<0.001	2.40	0.15	<0.001	11.64	1.06	<0.001	10.15	0.98	<0.001	8.54	0.98	<0.001	18.61	1.17	<0.001
Groups	0.74	0.32	0.024	0.65	0.21	0.002	4.56	1.37	0.001	2.96	1.35	0.030	3.23	1.23	0.009	−2.50	1.52	0.100
Binge days	0.03	0.01	0.022	−0.02	0.01	0.026	0.22	0.06	<0.001	0.10	0.05	0.062	0.10	0.05	0.062	−0.20	0.07	0.004
Groups × binge days	−0.02	0.02	0.278	0.01	0.01	0.487	−0.20	0.08	0.012	−0.10	0.07	0.159	−0.13	0.07	0.089	0.18	0.09	0.038

## 4 DISCUSSION

A measure of severity should provide information not only on the intensity of defining psychopathological features of the illness but also on the level of functional impairment and the need for treatment services. The latter, for example, is likely to be of interest to patients, clinicians, and insurance providers. Therefore, this study compared persons with BN as a function of purging frequency according to the DSM‐5 severity specifier on eating disorder‐specific and general psychopathology. In addition, we also examined the number of purging methods used as an alternative severity rating of BN, which has been suggested by previous studies (Gianini et al., 2017). Finally, we explored how binge‐eating frequency would relate to eating disorder‐specific and general psychopathology as a function of severity groups.

### 4.1 Differences between severity groups

Higher purging frequency related to higher eating disorder‐specific and general psychopathology in the current study, although life satisfaction did not differ between severity groups. Differences between severity groups based on number of purging methods used were less consistent in that they mainly differed in eating disorder‐specific variables but not in variables related to general psychopathology (except anxiety symptoms). Of note, patients using multiple purging behaviours showed more frequent vomiting, use of laxatives, and compulsive exercise than patients using one purging method only, indicating that the frequency of purging and the number of purging methods used cannot clearly be distinguished. While the current results are partially in line with other studies that documented higher eating disorder‐specific and general psychopathology associated with the use of multiple purging methods (Eddy et al., 2009; Edler et al., 2007; Favaro et al., 2005), it appears that purging frequency is somewhat better suited for indicating severity in persons with BN.

### 4.2 Correlates of binge‐eating frequency

When examining correlates of the number of binge days, it turned out that these only partially overlapped with the correlates of BN severity, that is, purging frequency or the number of purging methods used. Specifically, while more frequent binge eating related to higher eating disorder psychopathology, more depressive symptoms, and lower life satisfaction, more frequent binge eating related to less compulsive exercise. Thus, it appears that more frequent binge eating is not necessarily followed by more frequent or a higher number of compensatory behaviours. This dovetails with the finding that patients using multiple purging behaviours did not differ from patients using one purging method in the number of binge days, which may also explain why those with multiple purging behaviours had a lower BMI than those using one purging method only. Thus, it seems that—as both groups experienced binge eating equally often—using more than one purging method successfully led to weight loss.

### 4.3 Interactive effects between binge eating and BN severity

The current study not only assessed the correlates of BN severity and of binge‐eating frequency, but also tested interactive effects between BN severity and binge eating on eating disorder‐specific and general psychopathology. Although these interactive effects were not consistently found across the different BN severity classifications and dependent variables, the overall picture suggests that relationships between more binge days and higher eating disorder‐specific and general psychopathology are attenuated or even reversed in persons with a high purging frequency or in persons that use multiple purging methods. For example, more binge days related to higher eating disorder‐specific psychopathology in persons with infrequent purging but to lower eating disorder‐specific psychopathology in persons with frequent purging. This finding suggests that frequent purging may somewhat alleviate weight‐ and shape concerns but binge eating in the absence of regular purging intensifies them. Similarly, more frequent binge eating related to higher depressive symptoms in persons with infrequent purging and in persons using one purging method but not in persons with frequent purging and in persons using multiple purging methods. Again, we speculate that these relationships reflect that frequent purging or using multiple purging methods after binge‐eating episode is a relief in persons with BN, in line with findings showing that negative affect increases after binging but decreases after purging (Haedt‐Matt & Keel, 2011).

### 4.4 Limitations

Interpretation of the current findings is limited to day‐ and inpatients with BN treated in Germany and may not translate to outpatients or persons with BN who do not receive treatment and to patients with BN treated in countries with different healthcare systems. For example, patients with BN were diagnosed according to ICD‐10 (World Health Organization, 2019), which is current classification system used in Germany. Thus, it might be that not all patients would also have fulfiled the DSM‐5 diagnostic criteria. However, we would argue that this is very unlikely. In ICD‐10, BN is defined as a syndrome characterised by repeated bouts of overeating and an excessive preoccupation with the control of body weight, leading to a pattern of overeating followed by vomiting or use of purgatives. In addition, binging and purging should occur at least twice a week across at least 3 months and, thus, this criterion is similar to DSM‐IV (American Psychiatric Association, 1994). In DSM‐5, this criterion was lowered to a frequency of at least once per week and, thus, persons diagnosed according to ICD‐10 generally meet the DSM‐5 criteria as well, which is also reflected in studies showing that prevalences for BN do not differ when using the DSM‐IV or DSM‐5 criteria (Mancuso et al., 2015).

Furthermore, this was a cross‐sectional, questionnaire‐based study. Self‐report measures may potentially be biased due to retrospective recall, social desirability, or demand effects. Moreover, the cross‐sectional design of the study does not allow for drawing causal inferences about the direction of effects. Thus, future studies that assess these variables in real time (e.g., with ecological momentary assessment) or with diagnostic interviews are needed to replicate the current results. Such studies may also reveal temporal dynamics between core symptoms of BN (binge eating and purging) and the psychopathological variables examined in the current study, similar to findings from the affect regulation literature on BN (Alpers & Tuschen‐Caffier, 2001; Haedt‐Matt & Keel, 2011). These methods may also be helpful in establishing causal chains that link the investigated behaviours and cognitive‐affective variables.

Another consideration is that the current study only examined a restricted number of purging behaviours (i.e., self‐induced vomiting, use of laxatives, and excessive exercise) and, thus, results may differ when other purging behaviours would be considered as well. However, in a large sample of inpatients with eating disorders (Quadflieg et al., 2023), self‐induced vomiting (44%) and use of laxatives (25%) were the most commonly reported purging methods while less than 10% of the sample reported using diuretics, appetite suppressants, thyroid hormones, enemas, emetics, or skipping insulin injections (numbers based on a personal communication with the first author). Thus, it appears that other purging behaviours are rarely used, making it unlikely that including those in the current analyses would substantially change results.

Finally, we would like to highlight that we ran and report numerous inferential tests in this manuscript, which increases the risk of Type I error. That is, we considered effects as significant when *p* < 0.05 and did not correct this decision rule for multiple testing because this would have substantially increased the risk of Type II error (Witt, 2019). Thus, results of the current study require replication in other samples.

### 4.5 Conclusion

In conclusion, this study found that purging frequency and number of purging methods seems to go hand in hand but using purging frequency as an indicator for BN severity produces a more consistent pattern of associated eating disorder‐specific and general psychopathology. However, it appears that focussing only either on the frequency or on the number of purging behaviours may be too short‐sighted. Instead, results suggest that it is necessary to take the number of binge‐eating episodes into account when evaluating severity of BN as well. As the current guidelines suggest inpatient treatment for patients with BN when their purging behaviour seems intractable (Harrington et al., 2015; Herpertz et al., 2011), patients with an extremely high number of binge episodes might be overseen and miss out on suitable treatment settings, even though they have a pressure of suffering. Future studies need to focus on binge‐eating episodes and their resulting psychological strain, particularly when there is a divergence of binge eating and purging (e.g., when a person binges without subsequent purging or purges without having previously binged).

## Data Availability

The data that support the findings of this study are available from the corresponding author upon reasonable request.
